# Identification of Protease Specificity Using Biotin-Labeled Substrates

**DOI:** 10.2174/1874091X01711010027

**Published:** 2017-04-21

**Authors:** Hiroyuki Yamamoto, Syota Saito, Yoshikazu Sawaguchi, Michio Kimura

**Affiliations:** 1 Department of Microbiology and Molecular Cell Biology, Nihon Pharmaceutical University, 10281 Komuro, Inamachi, Kitaadachi-gun, Saitama, 362-0806, Japan; 2 Department of Clinical Pharmaceutics, Nihon Pharmaceutical University, 10281 Komuro, Inamachi, Kitaadachi-gun, Saitama, 362-0806, Japan

**Keywords:** Protease specificity, Mass spectrometry, Biotin-labeled peptides

## Abstract

**Background:**

Proteolysis constitutes a major post-translational modification. For example, proteases regulate the activation or inactivation of various proteins, such as enzymes, growth factors, and peptide hormones. Proteases have substrate specificity, and protease expression regulates the specific and regional activation or inactivation of several functional proteins.

**Methods:**

We demonstrate a novel method for determining protease specificity through the use of MALDI-TOF mass spectrometry with biotin-labeled substrates.

**Results:**

This method was able to determine the specificity of TPCK-trypsin, V8 protease, elastase and cyanogen bromide cleavage, and the results were similar to previous reports. In addition, the method can be used to measure crude samples, such as tumor extracts.

**Conclusion:**

We demonstrated that this method could identify protease specificity after simple processing, even for crude samples.

## INTRODUCTION

Most proteins are activated by post-translational modifications, such as glycosylation, phosphorylation, and proteolysis. Proteolysis is related to the regulation of protein activities, such as for endocrine peptides and growth factors [1-4], protein activation (*e.g.*, for zymogen and proenzymes), intracellular protein digestion (*e.g.*, for lysosomes and proteasomes) [5, 6], and digestion of dietary protein intake [7].

One method of controlling the activity of proteins is through specific protease cleavage sites. Proteases recognize specific amino acid sequences and conformations of substrates and subsequently cleave them. Identification of this specificity is important for understanding the function and mechanism of proteases. Recently, some proteins with previously known functions showed novel physiological effects, for example, mitochondrial proteins and tenascin C. The mitocryptides-1, -2 and -CYC originate from mitochondrial cytochrome c oxidase subunit VIII, cytochrome b, and cytochrome c, respectively [8-10]; and these peptides contribute to the inflammatory response. The tenascin C fragments cleaved by matrix metalloproteinases induce cartilage disruption in arthritis [11]. In addition, protease inhibitors, such as the Bowman-Birk and Kunitz protease inhibitors chymostatin and antipain, suppress tumor growth [12]. Moreover, we have reported that during tumor growth and melanogenesis, some neuropeptides are released as in precursor forms, after which proteases activate the precursor in the extracellular milieu [13-15]. It is not yet clear exactly how the neuropeptides released in precursor form are activated [16-19]. Therefore, it is important to search for specific proteases in tissues to clarify how precursor peptides become activated.

Protease activity is frequently measured using synthetic substrates based on protease specificity determined by spectrometric assay (p-nitroanilide substrates, p-NA [20]) and fluorometric assays (coumarin substrates [21] and Fluorescence Resonance Energy Transfer, FRET [22]). p-NA and coumarin substrates are useful for measuring the “known specificity” of protease activities. In addition, FRET assays can obtain multiple types of information on protease function, including substrate interaction and recognition. Conversely, when studying protease specificity, spectrometric and fluorometric assays require multiple sequences and independent measurements, and even the determination of protease specificity for one amino acid requires 20 substrates and measurements.

Due to its sensitivity, selectivity, and speed, mass spectrometry is a useful method for identifying proteins, sugars, and nucleotides. Therefore, mass spectrometry has been previously used for many proteome and metabolome analyses. Mass spectrometry has also played an important role in the determination of specific protease cleavage sites using database searches of peptide libraries [23-26]. While these methods are powerful, their operation and analysis require experience and time.

Here, we described a novel method to identify protease specificity using Matrix Assisted Laser Desorption / Ionization-Time of Flight (MALDI-TOF) mass spectrometry.

## MATERIAL AND METHODS

### Methods

**Fig. (1):**
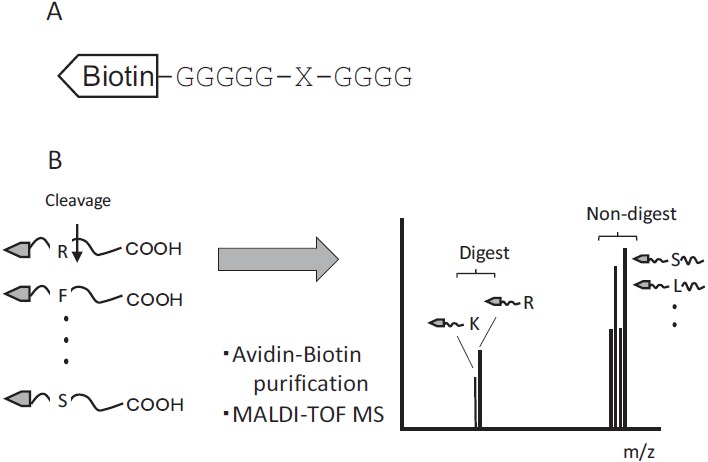
Schematic for identifying protease specificity A: Substrate structure, where the N-terminal amine is labeled with biotin and X indicates 20 amino acids. B: The procedure for determining protease specificity. Cleaved and non-cleaved substrates are purified with streptavidin-beads, after which the substrates are tested by MALDI-TOF mass spectrometry. Protease specificity is determined using the mass spectrometry results.

This method used a set of peptide mixtures in which each of the 20 amino acid residues was systematically substituted at the protease cleavage site. The N-terminals of peptides were labeled with biotin, and both the non-cleaved and cleaved peptides were purified with streptavidin-resin (Fig. **1A**). To distinguish between Leu and Ile, the N-terminal glycine spacer [-(Gly)_5_-] was elongated by an additional glycine residue [–(Gly)_6_-]. These peptides, as a mixture of substrates, were digested with samples containing proteases. The substrates were isolated from digests using the biotin-avidin affinity method. The isolated substrates were subjected to MALDI-TOF mass spectrometry, after which the molecular forms of cleaved fragments coincided with their theoretical molecular weights (Fig. **1B**).

### Peptide Synthesis

Peptides were synthesized by the Merrifield solid phase method manually with 9-fluorenylmethoxycarbonyl (fmoc) chemistry on 4-[2,4-dimethoxyphenyl-N-(9-fluorenylmethoxycarbonyl)aminomethyl]phenoxy resin [27]. After cleavage from resins, crude peptides were purified using an octadecyl silica (ODS) gel (SEPPAC-C18, Waters, MA). Crude peptides were bound to the ODS gel and washed with 0.1% trifluoroacetic acid (TFA). The peptides were eluted with 60% (v/v) acetonitrile in 0.1% TFA. The eluate was lyophilized and the purified peptide was weighed. The molecular weights of the synthetic peptides were determined using MALDI-TOF mass spectrometry (Autoflex, Bruker Daltonics, Germany). MALDI-TOF mass spectrometry was manually acquired in a positive reflect mode controlled by FlexControl software (Bruker Daltonics, Germany).

### Materials

N-tosyl-L-phenylalanine chloromethyl ketone (TPCK)-trypsin was purchased from Sigma–Aldrich (MO, USA). *Staphylococcus aureus* V8 proteases were obtained from Roche Diagnostics (Switzerland). Elastase was purchased from Worthington Biochemical Co. (USA). Peptide synthesis reagents were purchased from Watanabe Chemical Industries (Japan) and Tokyo Chemical Industry (Japan). Cyanogen bromide and formic acid were purchased from Wako Chemical (Japan).

### Animals

Male ICR mice were purchased from Japan SLC Co. Ltd (Shizuoka, Japan) and kept in SPF conditions in a temperature- and humidity-controlled room with a 12 h–12 h light–dark cycle. Mice were fed a normal diet and water *ad libitum*. All experimental protocols were approved by the Nihon Pharmaceutical University Laboratory Animal Care Advisory Committee and were in accordance with the guidelines of the US National Institutes of Health for the care and use of laboratory animals.

### Cell Cultures

The mouse sarcoma cell line S180 was obtained from the RIKEN Bio Resource Center (Ibaraki, Japan) and cultured in RPMI-1640 medium (Wako Pure Chemical Industries, Osaka, Japan) supplemented with 10% (v/v) fetal bovine serum (FBS, Biowest, France) in the presence of 5% (v/v) CO_2_ under 100% (v/v) humidity. When cultures achieved 80% confluency, the cells were dispersed with 0.25% trypsin and 0.02% ethylenediaminetetraacetic acid (EDTA) in phosphate-buffered saline (PBS) and harvested at an optimal concentration.

### Tumor Transplantation

S180 cells (1 × 10^6^ cells in 100 μl PBS or 100 μl PBS alone as a negative control) were implanted into ICR mice subcutaneously on the dorsal side. After tumors grew, mice were sacrificed and tumors were collected. The extracts of tumor proteins were prepared as follows: tumor tissue was homogenized in a threefold volume of 50 mM Tris-HCl buffer (pH 7.4) at 4°C and then centrifuged at 9,730×g at 4°C for 30 min. Supernatants were collected and stored at −20°C until use. The protein concentration of the extract was measured by the Coomassie Brilliant Blue G-50 method using bovine serum albumin (BSA).

### Digestion of Biotin-Labeled Substrates

10 μl of biotin-labeled substrates (final concentration, 5 ng/ml in Tris-HCl buffer) wasmixed with either 10 μl of enzymes (final concentration, 5 ng/ml) or tumor extracts (final concentration, 10 mg protein/ml) diluted with 50 mM Tris-HCl buffer (pH 7.4 for V8 protease or 8.0 for TPCK-trypsin and elastase). Negative controls were demonstrated using inactivated enzymes or tumor extracts, which were prepared by boiling for 10 min. The solution was then incubated at 37°C for 3 h, at which point the enzymatic digestion was stopped by incubation at 95°C for 10 min.

### Cyanogen Bromide Cleavage

10 μl of biotin-labeled substrates (final concentration, 5 ng/ml in 70% formic acid) were mixed with 10 μl of cyanogen bromide (CNBr) solution (final concentration, 1 mg/μl). The sample was incubated overnight at room temperature and then lyophilized. The digest was dissolved in 50 mM Tris-HCl buffer (pH 7.4) and processing to purify the biotin-labeled substrates.

### Purification of Biotin-Labeled Substrates

The digested biotin-labeled substrates were affinity-purified with streptavidin-Sepharose beads (GE Healthcare, UK). The biotin-labeled peptides were eluted by boiling for 10 min in 0.1% TFA. The eluted biotin-labeled peptides were applied to the plate and mixed with 1 μl of α-cyano-4-hydroxycinnamic acid (CHCA) solution or 2,5-dihydroxybenzoic acid (DHB) solution. Mass spectrometry experiments were carried out on a MALDI-TOF mass spectrometer (Autoflex, Bruker Daltonics, Germany). The mass spectrometry data were manually acquired in a positive reflect mode controlled by FlexControl software (Bruker Daltonics, Germany). The mass spectra were analyzed by FlexAnalysis software (Bruker Daltonics, Germany).

### SDS-PAGE

Tumor extracts were denatured with SDS and separated on 15% polyacrylamide gels [28]. The gels were visualized using Coomassie Brilliant Blue R250.

## RESULTS

### Tryptic Digestion

**Fig. (2):**
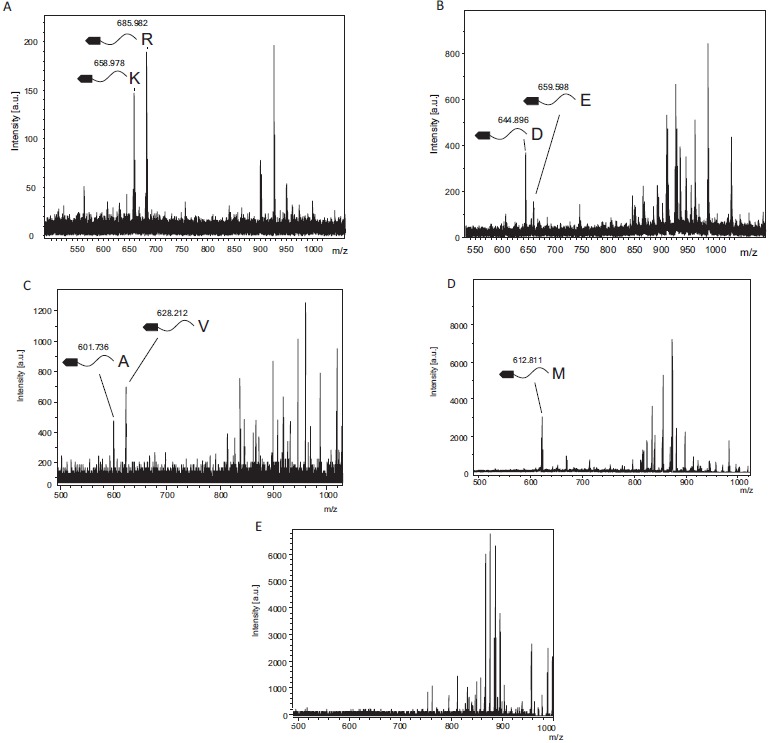
Protease specificity determination using model proteases and CNBr cleavage Mass spectra of trypsin, V8 protease, elastase and cyanogen bromide digestion. A: The fragments detected after cleavage of C-terminal Lys and Arg with tryptic digestion. B: The fragments generated from the cleavage of C-terminal Glu and Asp with V8 protease digestion. C: The fragments cleaved from the C-terminal after Ala and Val by elastase. D: The fragments generated from the cleavage of C-terminal Met after CNBr digestion. The mass spectra detected homoserine lactone converted from methionine during CNBr digestion. E: The biotin-labeled substrates treated with inactivated trypsin, which was boiled for 10 min, as a negative control.

TPCK-trypsin is known to cleave proteins at the carboxy side of lysine and arginine residues. We detected two fragments, 658.98 and 685.98, which coincided with fragments cleaved from the C-terminal after lysine (Theoretical m/z, 657.225) or arginine (Theoretical m/z, 685.2311), respectively (Fig. **2A**). In contrast, these cleaved fragments were undetectable using inactivated TPCK-trypsin, which was boiled before experiments (Fig. **2E**).

### V8 Protease Digestion

V8 protease was estimated to cleave proteins at the carboxy side of aspartic and glutamic acid residues. From Fig. (**2B**), we could detect two fragments, 644.89 and 659.60, which agreed with the theoretical values estimating the C-terminal cleaved after aspartic acid (Theoretical m/z, 644.1569) or glutamic acid (Theoretical m/z, 659.1726). In contrast, these cleaved fragments were undetectable using inactivated V8 protease, which was boiled before experiments.

### Elastase Digestion

Elastase was estimated to cleave proteins at the carboxy side of alanine, valine and isoleucine residues, and theoretical m/z of digests were 600.1671, 628.1984 and 699.2141, respectively. We could detect two fragments, 601.74, and 628.21 (Fig. **2C**), which matched theoretical m/z of alanine and valine, respectively. These digests were undetectable using inactivated elastase.

### Cyanogen Bromide Cleavage

Cyanogen bromide cleavage is a classical chemical cleavage method and cuts with a specific cleavage at the carboxy side of methionine residues. Methionine is then converted into homoserine or homoserine lactone, resulting in theoretical m/z of digests of 630.5181 and 612.5076, respectively. We could detect one fragments, 612.81 (Fig. **2D**), which matched the theoretical m/z of cleavage at the carboxy side of methionine and a subsequent modification to homoserine lactone.

### Protease Specificity Determination in Tumor Extracts From S180 Sarcomas

**Fig. (3):**
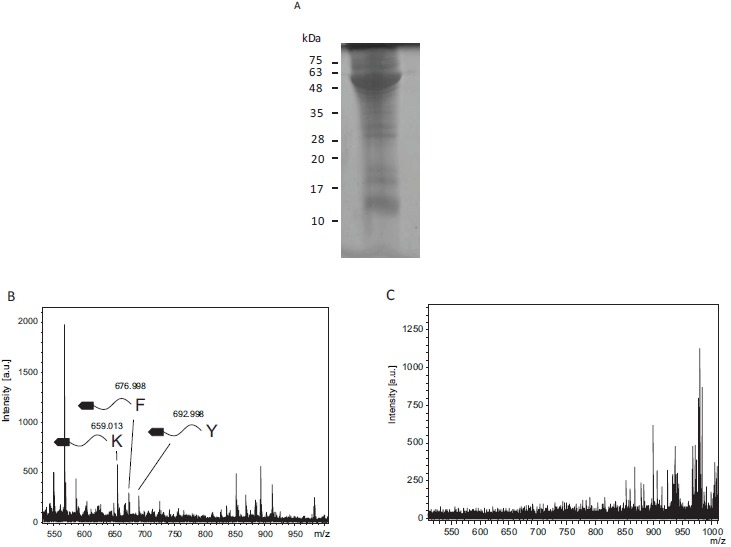
Protease specificity determination using tumor extract from S180 sarcomas A: SDS-PAGE analysis for the tumor extract. B: Mass spectra of the biotin-labeled substrates digested with tumor extracts showing the fragments detected after cleavage of C-terminal Lys, Phe, and Tyr. C: Mass spectra of the biotin-labeled substrates digested with tumor extracts inactivated by boiling for 10 min.

Tumor extracts were loaded into SDS-PAGE and stained by Coomassie Brilliant Blue R250 (Fig. **3A**). The tumor extract contained many proteins. Proteases in the tumor extracts were tested to determine protease specificity (Fig. **3B**). Cleaved fragments were detected at 659.01, 677.00, and 693.00, and were estimated to be fragments cleaved from the C-terminal after lysine (Theoretical m/z, 657.225), phenylalanine (Theoretical m/z, 676.1984), and tyrosine residues (Theoretical m/z, 692.1933), respectively. Conversely, when using tumor extracts after incubation in boiling water, these fragments were undetectable (Fig. **3C**).

## DISCUSSION

Proteases play an important role in several physiological functions, so the identification of their specificity is important for understanding the function and mechanism of proteases. Various methods for the identification of protease specificity have been developed, with corresponding advantages and disadvantages.

The current methods for determining protease specificity are summarized in Table **1**. Initially, protease specificity was predicted by collecting the results from native protein cleavage sites. This information is currently being accumulated in the MEROPS database (http://merops.sanger.ac.uk) [29], which then provides cleavage frequency by proteases. The MEROPS is a useful tool for determining “known” protease specificities. But the information of protease specificity, coming from relatively few reports, has low reliability. In addition, the determination of enzyme activity and specificity of restricted sequences can be done through direct photometric or fluorometric assays. In particular, coumarin substrates are frequently used for determining protease specificity due to their ease of use and high sensitivity. The direct photometric and fluorometric assays can also measure protease activity using only photometry or fluorometry, thus making them useful tools to reveal protease properties. Conversely, some proteases recognize several amino acids. The FRET assay is used for measuring such proteases, as it is able to measure protease activity and specificity. Several suppliers sell peptide libraries for determining protease specificity, although this is an expensive option. Recently, several methods have begun using proteomics, where proteome data is used to identify cleaved peptides by tandem mass spectrometry to determine cleavage sites [23-26]. This method, which requires operator experience, revealed specific N-terminal cleavage sites at different amino acids and showed the specificity of several proteases, such as Factor Xa [30], six type II transmembrane serine proteases [31], and membrane-type 6 matrix metalloprotease [32]. More recently, it has been reported that the substrate specificity of any endo- or exo-peptidase can be determined using LC-MS/MS sequencing along with multiple peptide libraries, a method termed MSP-MS [33]. In addition, MSP-MS can also measure protease activity in a single experiment. However, it is difficult to use MSP-MS to identify protease specificities from crude samples.

In this report, we showed a novel method performed through MALDI-TOF mass spectrometry using biotin-labeled substrates. Using TPCK-trypsin and V8 protease as model proteases, we were able to determine protease specificity from biotin-labeled substrates, as expected from previous reports. In addition, cleaved fragments were undetectable after protease inactivation. These results indicate that the appearance of fragments with low molecular weights was caused by specific protease cleavage. Furthermore, this new method can be used to analyze crude samples. In this study, we could determine the specificities of proteases within tumor extract derived from S180 sarcoma. While there were many proteins in the tumor extract, the biotin-labeled substrates could be purified by avidin-biotin affinity methods to be measured. However, while MALDI-TOF can show specific cleavage, it cannot measure the amount of protease activity.

In conclusion, we demonstrated that this novel method could be used to identify protease specificity conveniently and inexpensively, even for crude samples.

## Figures and Tables

**Table 1:** Methods for determining protease specificity ◎ means excellent, “○” means good, “△” means fair and “×” means poor.

	MEROPS database	Photometric or Fluorometric assay	FRET assay	Proteome-based method	Biotin-labeled substrate
Sensitivity	-	○	○	○	○
Specificity	◎	○	◎	◎	○
Cost	◎	△	×	×	△
Easily	◎	○	○	×	○
Crude sample	-	×	×	×	◎
Activity	×	◎	◎	△	×

## LIST OF ABBREVIATIONS

FRET: = Fluorescence Resonance Energy Transfer,
MALDI-TOF: = Matrix Assisted Laser Desorption / Ionization-Time of Flight, fmoc: 9-fluorenylmethoxycarbonyl,
TFA: = Trifluoroacetic acid, ODS: octadecyl silica,
TPCK: = N-tosyl-L-phenylalanine chloromethyl ketone,
CNBr: = Cyanogen bromide,
CHCA: = α-Cyano-4-hydroxycinnamic acid,
DHB: = 2,5-dihydroxybenzoic acid,
SDS-PAGE: = Sodium dodecyl sulfate-Poly Acrylamide Gel Electrophoresis
